# AFP-LSE: Antifreeze Proteins Prediction Using Latent Space Encoding of Composition of k-Spaced Amino Acid Pairs

**DOI:** 10.1038/s41598-020-63259-2

**Published:** 2020-04-28

**Authors:** Muhammad Usman, Shujaat Khan, Jeong-A Lee

**Affiliations:** 1 Department of Computer Engineering, Chosun University, Gwangju, 61452 Republic of Korea; 2 Department of Bio and Brain Engineering, Korea Advanced Institute of Science and Technology (KAIST), Daejeon, 34141 Republic of Korea

**Keywords:** Computational biology and bioinformatics, Machine learning, Computer science

## Abstract

Species living in extremely cold environments resist the freezing conditions through antifreeze proteins (AFPs). Apart from being essential proteins for various organisms living in sub-zero temperatures, AFPs have numerous applications in different industries. They possess very small resemblance to each other and cannot be easily identified using simple search algorithms such as BLAST and PSI-BLAST. Diverse AFPs found in fishes (Type I, II, III, IV and antifreeze glycoproteins (AFGPs)), are sub-types and show low sequence and structural similarity, making their accurate prediction challenging. Although several machine-learning methods have been proposed for the classification of AFPs, prediction methods that have greater reliability are required. In this paper, we propose a novel machine-learning-based approach for the prediction of AFP sequences using latent space learning through a deep auto-encoder method. For latent space pruning, we use the output of the auto-encoder with a deep neural network classifier to learn the non-linear mapping of the protein sequence descriptor and class label. The proposed method outperformed the existing methods, yielding excellent results in comparison. A comprehensive ablation study is performed, and the proposed method is evaluated in terms of widely used performance measures. In particular, the proposed method demonstrated a high Matthews correlation coefficient of 0.52, F-score of 0.49, and Youden’s index of 0.81 on an independent test dataset, thereby outperforming the existing methods for AFP prediction.

## Introduction

In Antarctic fish, a survival mechanism that prevented them from freezing in seawater at sub-zero temperatures was observed, which led to the discovery of antifreeze proteins (AFP)^1^. AFPs have been identified as a crucial substance for resisting a freezing environment in various species including plants, bacteria, fungi, insects, and animals^2^. Ice exists in different geometric shapes due to the varying arrangements of oxygen atoms; therefore, the structural and sequential arrangements of AFPs largely vary to accommodate this heterogeneity of ice molecules^3^. Ice also exhibits the property of recrystallization, by which small ice crystals bind to the water molecules, thus becoming a large ice lattice, causing severe damage to the cell membrane, which, in some cases, may be lethal^4^. AFPs are commonly categorized into glycoproteins (AFGPs) and non-glycoproteins (AFPs)^5^. They protect the organisms using two mechanisms: (i) thermal hysteresis (TH), by which the freezing point of water is depressed to a few degrees by the adsorption-inhibition effect without altering the melting point^6^; (ii) ice crystal inhibition, by which the AFP sites bind to the surfaces of ice and inhibit their growth to become a larger ice lattice, developing either small harmless ice crystals or forming a needle-shaped lattice, thus diminishing the recrystallization property of ice^2^.

AFPs are indispensable in organisms such as fish^7^, fungi^8^, bacteria^9^, plants^10^, and insects^11^. Furthermore, they are essential in various medical applications (for example, cryopreservation and cryosurgery)^12^ and food industry^13^. The ice-binding mechanism of proteins is not fully understood^14^. Reliable prediction of AFPs may play a fundamental role in identifying the underlying ice-binding mechanism. Accurate prediction would lead to the understanding of protein-ice interaction, which in turn would enable the design of macro-molecular antifreeze proteins with enhanced efficiency^15^. Studies indicate that AFPs show minute or, in most cases, no similarity in structures, sequences, and ice-binding sites within closely related species^3,5,16,17^. For instance the sub-types of AFPs found in fishes namely Type I, II, III, IV and AFGP^15^, have no significant similarities in structures and sequences; rather, they demonstrate some homology to different protein families from which they are assumed to have evolved^18,19^. This inconsistency makes their in-silico identification using conventional search tools such as BLAST^20^ and PSI-BLAST^21^ unfavorable and increases the complexity of the development of a reliable prediction model due to the lack of common features.

Researchers have proposed several computational strategies such as machine learning to achieve superior results for this diversified classification problem. Kandaswamy *et al*. proposed a framework named AFP-Pred, which is considered to be a pioneering work in this direction, to utilize machine learning^22^. In this method, a feature vector containing 119 attributes was obtained by encoding each sequence, from which dominant features were selected using the ReliefF approach to train the random forest (RF) classifier. Yu *et al*. proposed a web-based predictor named iAFP^23^, which utilized n-peptide composition to obtain the feature set. Superior features were selected using the genetic algorithm, and the resultant features were employed to train a support vector machine (SVM). Xiaowei *et al*. used position-specific scoring matrix (PSSM) profiles with an SVM classifier to develop a web-based AFP predictor called AFP_PSSM^24^. Mondal *et al*. used the sequence order information from Chou’s pseudo amino acid composition (PseAAC) with an SVM to develop an algorithm for AFP prediction (AFP-PseAAC)^25^. Yang *et al*. developed an ensemble-based learning method named AFP-Ensemble^26^, in which the RF classifier was trained for predicting AFPs. As they performed the evaluation on a non-standard dataset, their results are not discussed in this study. Xiao *et al*. developed a predictor named iAFP-Ense^27^ by incorporating evolutionary information into PseAAC using RF classifiers; however, the classifier was not evaluated on an independent test dataset. Khan *et al*. performed segmentation of protein sequences to divide them into two groups for amino acid composition (AAC) and di-peptide composition analyses^28^. The dominating features were selected using information gain and ranker methods, and classification was performed using the RF classifier. A web-based predictor for AFPs called CryoProtect^29^ is proposed using the RF classifier. The predictor used AAC and di-peptide composition as features for the classifier. The classification of AFP from other protein families is an example of a class imbalance problem. A widely adopted technique to deal with the unbalanced dataset is resampling^30^. Simple resampling techniques involve over-sampling, in which records from the minority class are randomly duplicated, and under-sampling, which executes a random removal of some records from the majority class. However, over-sampling has been reported to pose the problem of overfitting^31^ and under-sampling leads to the loss of information^32^. To overcome these limitations Nath *et al*. adopted *K*-means clustering with ensemble prediction algorithms to predict AFPs^19^.

The aforementioned methods have shown a reasonable improvement in prediction performance. However, there is a need for an improved method to obtain the desired results. In particular, to the best of our knowledge, none of the methods discussed above have achieved a balanced accuracy value of 90% or above on the standard dataset.

In this work, we utilize the composition of *k*-spaced amino acid pairs (CKSAAP) for the numerical representation of the amino acid sequence, which has been successfully adopted by several researchers to address various prediction problems^33–35^. A part of this work was presented in^36^, where we explored the discrimination power of *k* = 0 to 13-spaced amino acid pairs. More specifically, we observed that a gap of *k* = 8 provides the best classification performance.

In recent times, deep learning has been used in various bio-informatics applications^37,38^. It has also been very successfully employed for classification problems^39^. The novelty of our work is that, for the first time, a deep-learning-based technique has been proposed for the classification of AFP sequences. As the dataset is significantly small in size and, with *k* = 8, the number of descriptors of the CKSAAP scheme is 3600, the training of the model becomes an ill-posed problem.

In this paper, we propose a novel machine-learning-based approach using the concept of latent space learning through a task-specific deep auto-encoder. An auto-encoder, generally used for feature compression^40^, is now utilized to perform composite functions, i.e., to extract significant features from the encoding scheme and to perform the prediction task. The auto-encoder is modified to learn minimally redundant and maximally relevant latent space features, and hence, the feature length is drastically reduced. Exploiting only these important attributes, the classifier achieves superior performance.

**Figure 1:**
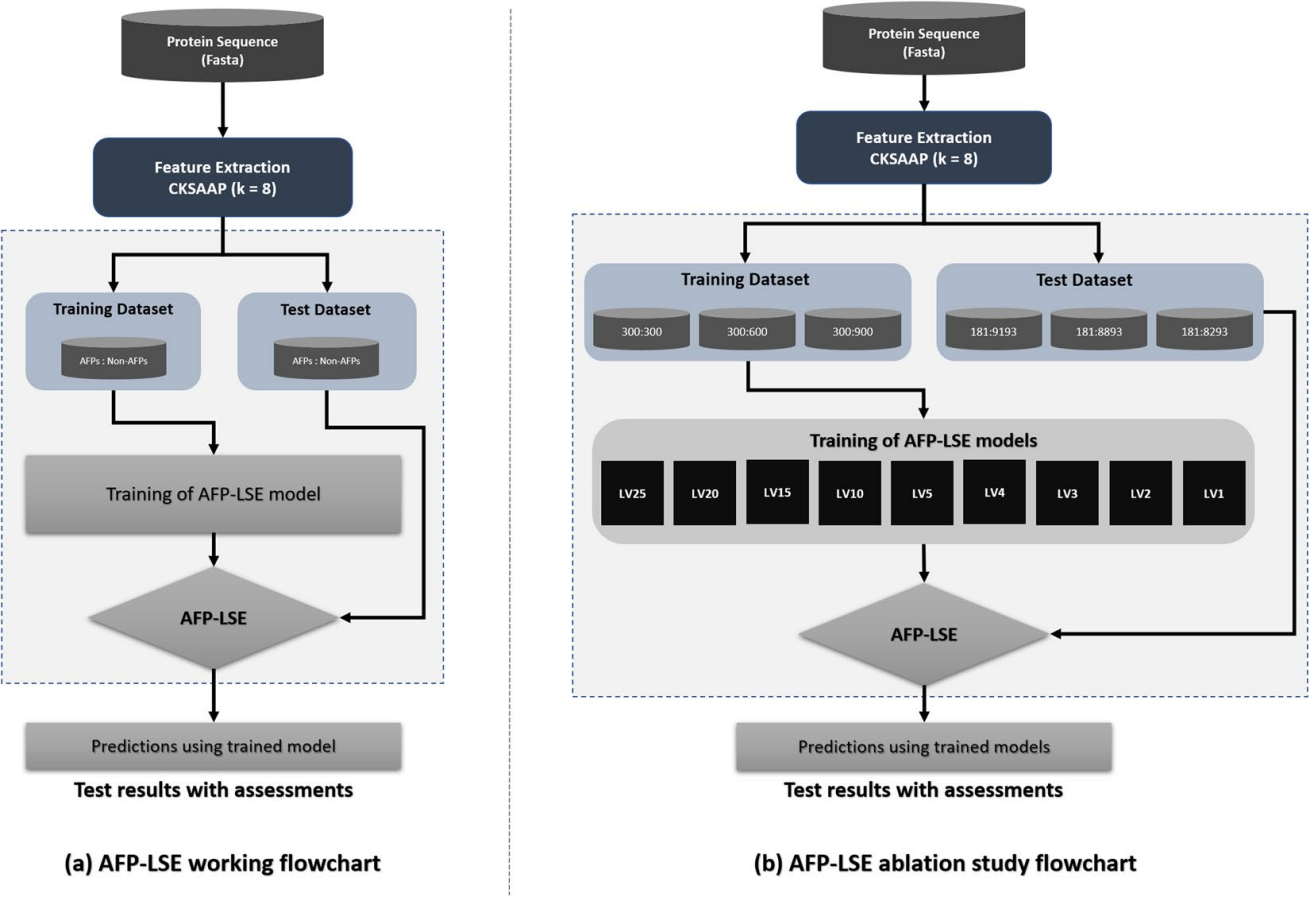
(**a**) Workflow of the proposed algorithm. The features are extracted using CKSAAP encoding scheme by keeping the gap value *k* = 8. (**b**) Workflow of the ablation studies. To perform the ablation studies, the dataset is divided into training and test sets, where training dataset is composed of 1:1, 1:2 and 1:3 AFP:Non-AFP ratios i.e., 300:300, 300:600 and 300:900 AFPs:Non-AFPs respectively and remaining samples were used for test dataset. For each case 9 different models of latent variable size (*LV* = 1, 2, 3, 4, 5, 10, 15, 20 and 25) were designed.

A thorough ablation study is performed on the model to obtain the optimal values of the hyperparameters and latent space size. The best model produces superior results on the evaluation parameters including the Matthews correlation coefficient (MCC), Youden’s index, balanced accuracy and F1 score. The workflow of the proposed method and the ablation studies performed are shown in Fig. 1, and its details are discussed in later sections.

## Methods

### Evaluation parameters

AFP prediction is considered a classification problem. Accordingly, we use standard threshold-dependent parameters including sensitivity, specificity, accuracy, MCC, balanced accuracy, Youden’s index and F1 score to evaluate the performance of the proposed classifier. These parameters can be evaluated using the following equations:

1 $$Sensitivity=\frac{TP}{TP+FN}$$

2 $$Specificity=\frac{TN}{TN+FP}$$

3 $$Accuracy=\frac{TP+TN}{TP+TN+FP+FN}$$

4 $$MCC=\frac{TPTN-FPFN}{\sqrt{(TP+FP)(TN+FN)(TP+FN)(TN+FP)}}$$

5 $$Balanced\,Accuracy=\frac{Sensitivity+Specificity}{2}$$

6 $$Youden{\prime} s\,Index=Sensitivity+Specificity-1$$

7 $$F1\,Score=2\ast \frac{{Precision}\ast {Recall}}{{Precision}+{Recall}}$$

8 $${Precision}=\frac{TP}{TP+FP}$$

Here TP, FP, TN, and FN represent true positive (correctly classified AFP), false positive (incorrect classification of non-AFP as AFP), true negative (correctly classified non-AFP), and false negative (incorrect classification of AFP as non-AFP), respectively. Thus, sensitivity indicates the fraction of AFPs correctly classified as AFPs and specificity indicates the fraction of non-AFPs correctly classified as non-AFP. Accuracy indicates the ratio of the total number of correctly classified samples to the total number of samples. As the test dataset is highly imbalanced, the parameters that assess the predictor’s quality considering the imbalanced distribution of the test data must be emphasized. For example, MCC considers the TP, TN, FP, and FN values and is regarded as a balanced measure, even if the test dataset is imbalanced. The range of MCC lies between −1 → 1, with −1 indicating the worst binary classification and 1 indicating the best binary classification. Furthermore, balanced accuracy, which is defined as an average of the recall obtained on each class, is usually used when the test dataset is imbalanced. Youden’s index is a class-specific measure, and the F-score represents the harmonic mean of precision and recall/sensitivity.

### Dataset

The benchmark dataset^22^ is obtained to assess the performance of our approach. The dataset was constructed by initially obtaining 221 AFPs from the Pfam database as seed. A stringent threshold, (*E* = 0.001), was chosen during the PSI-BLAST to remove any redundancy from the data. A manual check was performed to remove any non-AFPs, and finally, the CD-HIT program was used to reduce the sequence identity to 40%. The total number of proteins in the positive dataset is 481. The negative dataset has 9493 non-AFPs, which do not have overlap with the AFPs. These positive and negative datasets were divided into two subsets for training and testing.

For a fair comparison, the subsets are maintained to be quantitatively equal to the subsets used in the previous approaches i.e., 300 AFPs and 300 non-AFPs in the training subset, and 181 AFPs and 9193 non-AFPs in the test subset. The selection of proteins from the dataset was randomized to ensure generalization. Some methods have utilized an imbalanced training dataset to investigate the influence of the number of non-AFPs on the prediction performance^41^. Therefore, to determine the effect of data distribution, we performed an ablation study with 600, 900, and 1200 negative training samples during training while maintaining a constant number of positive samples i.e., 300.

### Features extraction

#### Composition of k-spaced amino acid pairs

Several machine-learning approaches have been utilized to perform the prediction task for AFPs^28,42^. The fundamental task in developing a computation-based classification model is the translation of protein sequences to interpretative encoded numerical features. Therefore, the conversion of sequence into the numerical vector is indispensable. Various encoding schemes that employ numerous protein features have been developed to extract diverse information from the protein sequences. As it was believed that an individual feature extraction strategy may only represent a partial target’s knowledge^26^, in numerous studies, multiple feature extraction methods are combined to enhance the classification performance^23,24,26,27^. However, it has been observed in recent studies that a viable feature extraction method e.g., CKSAAP can equally contribute toward satisfactory prediction performances^43–45^. Thus, we utilized CKSAAP encoding scheme in the AFP-CKSAAP method^36^.

**Figure 2:**
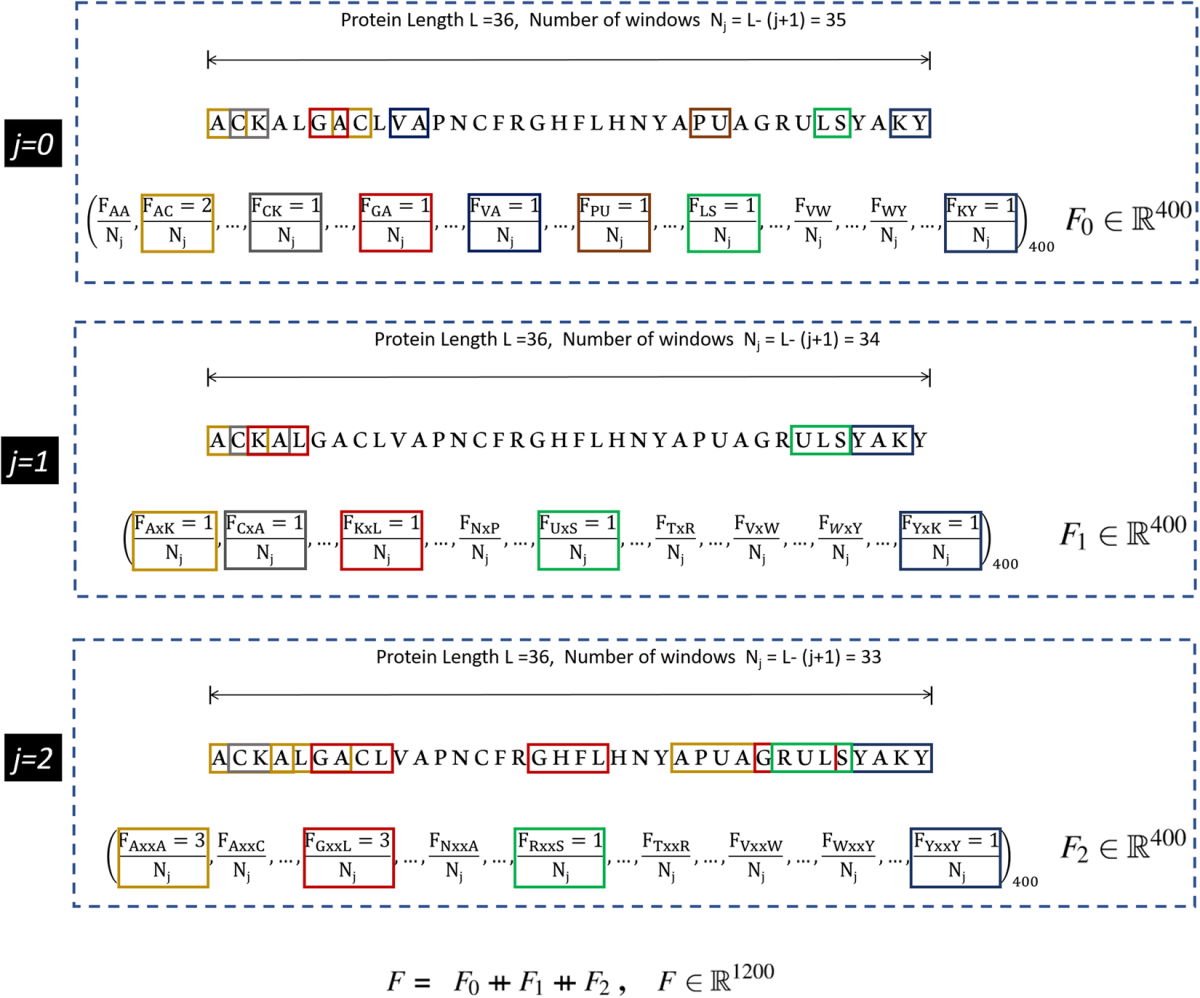
Illustration of CKSAAP descriptor calculation for *k* = 2.

This encoding method has emphasized the significance of amino acid pairs and has been utilized in various classification methods^34,35,46^. The feature vector is obtained by calculating the frequency of amino acid pairs separated by *k* (*j* = 0, 1, 2, … *k*) number of residues. The representation is based on the frequency of *k*-spaced amino acid pairs in a local sequence window. If *k* = 2, *k*-spaced pairs for *j* = 0, 1, and 2 are considered. For each value of *j*, the corresponding feature vectors *F*_*j*_ i.e., *F*_0_, *F*_1_ and *F*_2_ as shown in Eqs. (9), (10), and (11), respectively, are evaluated, each having a length of 400. The final feature vector *F* is computed by concatenating the individual feature vectors as shown in Eq. (12). The value of each descriptor is calculated by dividing the number of occurrences of that amino acid pair by the total number of *j*-spaced residue pairs (*N*_0_, *N*_1_ … *N*_*j*_) in the protein. For *j*, *N*_*j*_ = *L* − (*j* + 1), where *L* is the length of the protein sequence. In Fig. 2, only a few windows have been highlighted for the purpose of illustration. However, in practice, all the amino acid pairs are covered in overlapping windows with the respective gap values.

9 $${F}_{0}={\left(\frac{{F}_{AA}}{{N}_{0}},\frac{{F}_{AC}}{{N}_{0}},\frac{{F}_{AD}}{{N}_{0}},\ldots ,\frac{{F}_{YY}}{{N}_{0}}\right)}_{400}$$

10 $${F}_{1}={\left(\frac{{F}_{AxA}}{{N}_{1}},\frac{{F}_{AxC}}{{N}_{1}},\frac{{F}_{AxD}}{{N}_{1}},\ldots ,\frac{{F}_{YxY}}{{N}_{1}}\right)}_{400}$$

11 $${F}_{2}={\left(\frac{{F}_{AxxA}}{{N}_{2}},\frac{{F}_{AxxC}}{{N}_{2}},\frac{{F}_{AxxD}}{{N}_{2}},\ldots ,\frac{{F}_{YxxY}}{{N}_{2}}\right)}_{400}$$

12 $$F={F}_{0}+\,+{F}_{1}+\,+\ldots +\,+{F}_{j}+\,+\ldots +\,+{F}_{k},\,F\in {{\mathbb{R}}}^{400\ast (k+1)}$$

It is evident from Eq. (12) and Fig. 2, that the CKSAAP encoding scheme utilizes the the trivial information from the preceding features including AAC, DPC, and TPC, which have been proven to play a vital role in AFP prediction in earlier studies^22,28,29^.

#### Incremental feature selection

Selection of key representative parameters is important for improving the prediction performance of a classifier. AFP-CKSAAP has been thoroughly evaluated to determine the optimal value of *k* by manually performing the sequential forward selection method to determine the best-suited feature. The best performance of the classifier was obtained by maintaining the gap value *k* = 8^36^. It is also evident from the references that an attribute vector obtained from a very large value of *k* will include redundant features and may not contribute toward prediction^33,47^. Owing to the significance of maintaining this value of *k*, in this study, we perform all the performance analyses by maintaining the constant gap value of *k* = 8.

From Eq. (12), it can be inferred that the gap value *k* = 8 in CKSAAP retrieves a feature vector of length 3600. In AFP-CKSAAP, we utilized all the features for classification using a deep neural network that produced satisfactory results, outperforming the previously proposed methods by a fair margin. However, by training the algorithm with fewer training samples having large feature dimensions, there exists a possibility that the AFP-CKSAAP algorithm may lose its generalization for new samples. Therefore, in this study, we intend to achieve satisfactory prediction using a reduced number of features. This could be done by dimension reduction using existing methods such as principle component analysis^48^, Gini index^49^, and mutual information^50^. However, recently, an auto-encoder has also been effectively used for dimension reduction^51,52^. An auto-encoder, which is an unsupervised algorithm, has emerged as a successful neural network framework that learns to represent the input data in much fewer dimensions and regenerates an output approximately similar to the input that has been fed to it. The principal function of this algorithm is its ability to reconstruct the input using substantially fewer features by constraining the latent space. The properties of the latent space in the auto-encoder make it a favorable candidate for feature compression in this study. The details of the architecture of the auto-encoder and its utilization in this study are discussed later sections.

### Latent space learning for AFP classification

**Figure 3:**
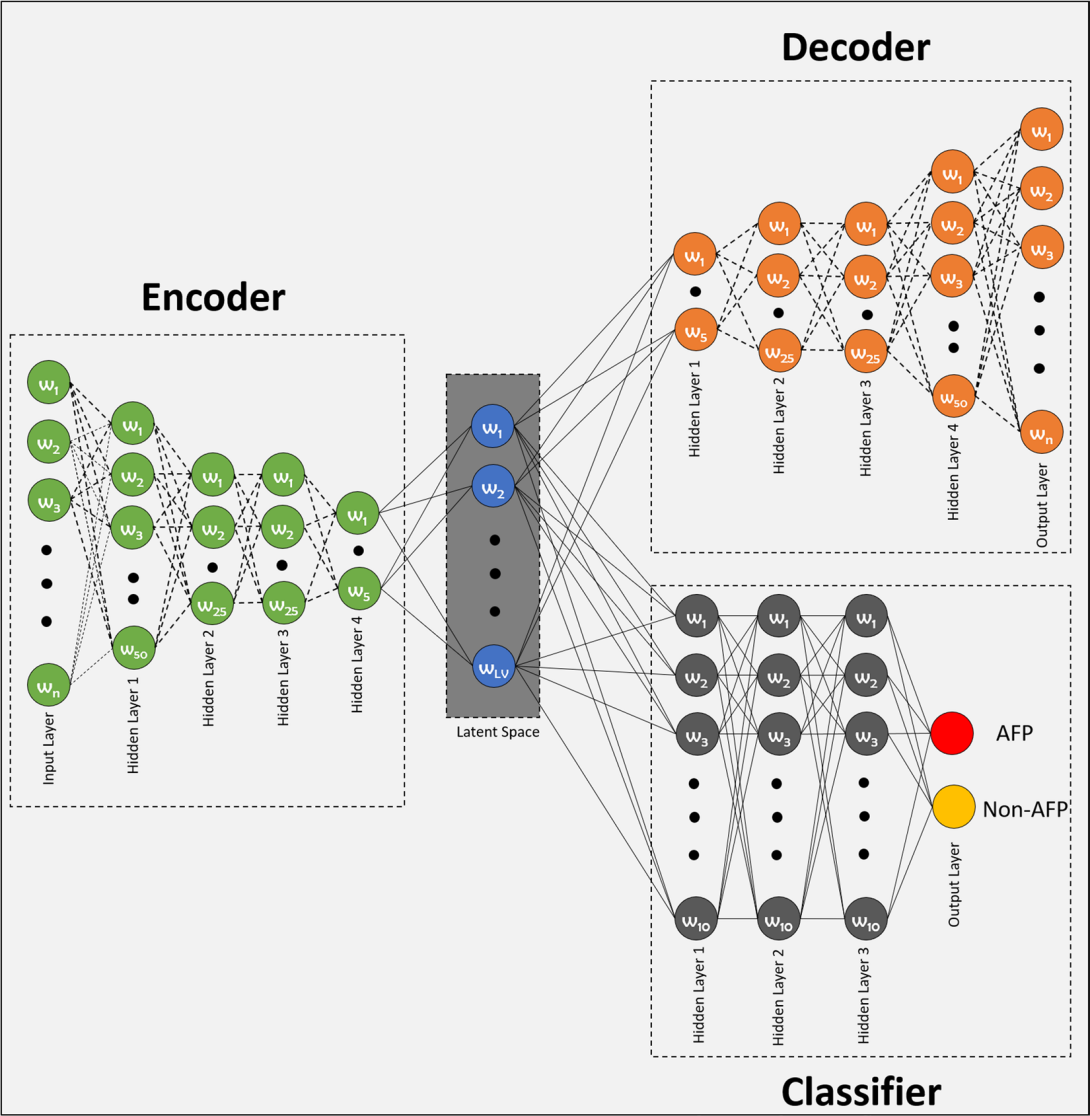
Architecture of the proposed model for AFP classification. The encoder is composed of an input layer and four hidden layers and embeds the observation to the latent space. The output layer of the encoder is the latent space, connected to the last hidden layer of the the encoder, and serves as the input for the decoder and classifier.The decoder is the complement of the encoder and decodes the representation to the original space. The classifier is a fully connected four-layered multilayer perceptron and is tuned to perform prediction task.

In this study, we design a novel auto-encoder-based classification model for the prediction of AFP proteins. The proposed model is a combination of auto-encoder and classifier. By simultaneously training the auto-encoder and classifier, we successfully learned a noise-free latent space representation, which is composed of variables that have learned the least redundant and most relevant attributes of the input data. The architecture of the proposed model is shown in Fig. 3.

#### Network specifications

##### Auto-encoder

An auto-encoder is an unsupervised learning algorithm that aims to learn to reproduce the input using fewer dimensions. We propose to use a multilayer auto-encoder architecture that has been regularized to be sparse to generate compressed latent space. By imposing a sparsity penalty during training, the model learns the most informative and discriminative features for AFP classification  from the input data as a byproduct^40^. The architecture is composed of three sections: (i) an encoder with some hidden layers, (ii) a latent space, which represents the encoded input in reduced dimensions by ignoring the noise in the input^53^, and (iii) a decoder that regenerates the input from the latent space variables. The number of hidden layers and the number of neurons in each layer of the encoder and decoder are varied to obtain reasonable performance. In this study, the encoder and decoder are composed of five layers, including four hidden layers. The number of neurons in the input layer of the encoder is equal to the length of the attribute vector, the number of neurons in the first hidden layer is 50, the numbers of neurons in the second and third hidden layers of the encoder are 25 each, and the fourth hidden layer has 10 neurons. The number of neurons in the latent space is systematically altered to obtain the best performance. The best performance was achieved when four neurons in the space were selected. The decoder is a complement of the encoder, this symmetry ensures the smooth encoding and decoding procedure^54^. Therefore, the number of neurons in the first hidden layer of the decoder is equal to that in the last layer of the encoder and so on i.e., the numbers of neurons in the first, second, third and fourth hidden layers of the decoder are 10, 25, 25, and 50 respectively. Finally, the number of neurons in the output layer of the decoder is equal to the length of attribute vector.

The latent space, represents the learned representative features, and is the middle layer of the auto-encoder. It is shared between the encoder and decoder, serving as the final layer for the encoder and the input layer for the decoder. In the proposed model, the latent space has been regularized to be sensitive to the unique statistical features of the input by adding a regularization term in the loss function.

Therefore, the model retrieves the information by using the most discriminative features only, essentially serving the classification task. Thus, the classifier is trained on the dominant features, and the decoder is trained to regenerate the input from the latent variables.

##### Classifier

The classifier is designed to process the latent space variables generated by the auto-encoder module. For the classification, a similar approach as in AFP-CKSAAP^36^ i.e., multilayer perceptron (MLP), is implemented. The architecture of the classifier, as shown in Fig. 3, is composed of three hidden layers and an output layer. The final layer of the encoder, which is the latent space, serves as an input layer for the classifier. Therefore, the input layer of the classifier has 4 neurons, each hidden layer has 10 neurons, and the number of neurons in the output layer is equivalent to the number of classes.

#### Training method

The model consisting of two modules, the auto-encoder module and the classifier module as shown in Fig. 3, is trained using Python on Keras (Tensorflow) for 1000 epochs with a variant of the gradient descent algorithm called Rmsprop^55^. Each layer of the auto-encoder module uses a rectified linear unit (ReLU) as an activation function to avoid a vanishing gradient. Furthermore, a dropout layer with 30% is used after each layer for better generalization and to avoid overfitting. For the classification module, ReLU has been used as an activation function for all the layers, except the output layer where the softmax function is used to generate class prediction probabilities.

The proposed model generates two types of outputs: (i) a decoded feature vector, and (ii) a class label of input protein. For the auto-encoder and classifier modules, we used different loss functions to minimize their respective error values. To train the auto-encoder, we use a mean squared error (MSE) loss function, whereas the classifier module is optimized by minimizing the binary cross entropy between the true class and predicted class labels. The MSE is calculated between the input and decoded feature vectors of the auto-encoder. The results of MSE values for all the auto-encoder models are presented in Table 1.

**Table 1:** Performance of the proposed method evaluated on widely used metrics for different data distributions and variations in the latent space size.

No. of latent variables:	LV1	LV2	LV3	LV4	LV5	LV10	LV15	LV20	LV25
Training samples ratios:	1:1 AFP:NON-AFP								
*Sensitivity (%)*	84.83 ± 3.95	79.72 ± 7.22	82.59 ± 6.39	82.18 ± 5.21	85.58 ± 5.40	82.59 ± 4.68	82.03 ± 5.50	81.13 ± 8.94	80.49 ± 6.94
*Specificity (%)*	91.52 ± 3.04	90.82 ± 4.39	89.95 ± 2.66	92.86 ± 4.01	88.95 ± 3.75	90.73 ± 3.25	93.06 ± 2.26	91.97 ± 3.99	91.51 ± 2.94
*Balanced Accuracy (%)*	88.17 ± 1.19	85.27 ± 2.63	86.27 ± 2.30	87.52 ± 1.40	87.26 ± 1.79	86.66 ± 2.01	87.54 ± 1.90	86.55 ± 2.78	86.00 ± 2.40
*Youden’s Index*	0.76 ± 0.02	0.70 ± 0.05	0.72 ± 0.04	0.75 ± 0.02	0.74 ± 0.03	0.73 ± 0.04	0.75 ± 0.03	0.73 ± 0.05	0.72 ± 0.04
*MCC*	0.46 ± 0.05	0.33 ± 0.04	0.32 ± 0.03	0.48 ± 0.07	0.32 ± 0.04	0.34 ± 0.06	0.48 ± 0.04	0.46 ± 0.06	0.34 ± 0.04
*F1-Score*	0.42 ± 0.07	0.26 ± 0.05	0.25 ± 0.04	0.46 ± 0.09	0.24 ± 0.05	0.27 ± 0.07	0.45 ± 0.05	0.43 ± 0.08	0.28 ± 0.06
*MSE (dB)*	−14.69 ± 3.34	−15.90 ± 4.08	−16.57 ± 5.30	−18.45 ± 6.40	−17.08 ± 7.45	−18.31 ± 7.72	−16.27 ± 2.26	−17.86 ± 4.92	−15.96 ± 4.42
**Training samples ratios:**	**1:2 AFP:NON-AFP**								
*Sensitivity (%)*	79.77 ± 7.69	75.74 ± 4.81	76.79 ± 7.06	83.42 ± 5.50	77.23 ± 7.50	77.73 ± 5.77	79.22 ± 8.14	82.04 ± 8.10	76.96 ± 8.10
*Specificity (%)*	93.16 ± 2.80	94.84 ± 1.33	94.08 ± 2.59	90.02 ± 4.38	93.23 ± 4.88	94.56 ± 2.40	93.29 ± 2.89	92.88 ± 2.50	94.21 ± 2.24
*Balanced Accuracy (%)*	86.47 ± 2.69	85.29 ± 2.11	85.43 ± 2.58	86.72 ± 1.25	85.23 ± 1.80	86.15 ± 1.72	86.26 ± 2.94	87.46 ± 1.42	85.58 ± 3.08
*Youden’s Index*	0.72 ± 0.05	0.70 ± 0.04	0.70 ± 0.05	0.73 ± 0.02	0.70 ± 0.03	0.72 ± 0.03	0.72 ± 0.05	0.74 ± 0.02	0.71 ± 0.06
*MCC*	0.38 ± 0.05	0.40 ± 0.03	0.40 ± 0.05	0.33 ± 0.05	0.39 ± 0.07	0.42 ± 0.06	0.38 ± 0.05	0.38 ± 0.05	0.40 ± 0.05
*F1-Score*	0.32 ± 0.08	0.36 ± 0.04	0.35 ± 0.07	0.26 ± 0.07	0.35 ± 0.10	0.37 ± 0.09	0.33 ± 0.07	0.32 ± 0.06	0.35 ± 0.07
*MSE (dB)*	−16.71 ± 6.38	−17.28 ± 4.30	−14.28 ± 2.38	−18.63 ± 4.54	−16.00 ± 3.05	−18.43 ± 4.99	−14.48 ± 2.46	−18.24 ± 2.79	−16.76 ± 4.72
**Training samples ratios:**	**1:3 AFP:NON-AFP**								
*Sensitivity (%)*	71.27 ± 2.60	80.11 ± 6.09	76.46 ± 6.15	75.74 ± 10.78	76.40 ± 5.13	76.68 ± 4.60	82.70 ± 4.97	76.62 ± 5.26	77.01 ± 7.26
*Specificity (%)*	94.77 ± 3.01	94.38 ± 2.84	96.21 ± 1.80	95.41 ± 2.23	95.19 ± 1.53	95.57 ± 1.67	93.28 ± 2.87	95.47 ± 1.90	95.84 ± 2.00
*Balanced Accuracy (%)*	83.02 ± 11.92	87.24 ± 1.94	86.33 ± 2.23	85.57 ± 4.63	86.30 ± 1.89	86.13 ± 1.75	87.99 ± 1.21	86.05 ± 1.79	86.42 ± 2.87
*Youden’s Index*	0.66 ± 0.23	0.74 ± 0.03	0.72 ± 0.04	0.71 ± 0.09	0.72 ± 0.03	0.72 ± 0.03	0.75 ± 0.02	0.72 ± 0.03	0.72 ± 0.05
*MCC*	0.37 ± 0.13	0.43 ± 0.07	0.48 ± 0.06	0.44 ± 0.05	0.47 ± 0.05	0.44 ± 0.06	0.40 ± 0.04	0.44 ± 0.06	0.46 ± 0.05
*F1-Score*	0.33 ± 0.13	0.38 ± 0.09	0.44 ± 0.08	0.40 ± 0.07	0.44 ± 0.07	0.41 ± 0.08	0.34 ± 0.06	0.40 ± 0.08	0.43 ± 0.07
*MSE (dB)*	−18.82 ± 7.91	−17.16 ± 3.86	−15.86 ± 2.51	−16.18 ± 3.82	−16.68 ± 1.85	−15.44 ± 2.21	−17.89 ± 4.84	−16.27 ± 3.60	−17.32 ± 2.87

## Results

Herein, we present the results of the experiments performed for the evaluation of the model. The training dataset is randomly divided into two subsets, i.e., training and validation, with the ratio of 90:10, i.e., out of 600 samples, 540 samples were used for training and 60 samples for validation. We used early stopping with the patience of 50 epochs to avoid overfitting, and we stopped the training if the model stopped improving. The metric in the early stopping was validation loss, and the training was stopped at approximately 700 epochs. The best model was obtained by performing the ablation study, the details of which are discussed later in the text.

### Ablation study

In this work, we perform an ablation study to obtain a simple overall architecture. This is motivated by the fact that the latent space is sparsely populated. This sparse space eliminates redundancies to achieve the degree of compression factor that can be reached. To this end, a benchmark architecture is evaluated with various modifications in the design, and the performance of each model is observed. One must choose an optimal number of neurons in the latent space so that the feature vector is significantly reduced, and the decoder must be able to regenerate the input using these features. Furthermore, the latent space serves as the input layer of the classifier network, which makes it crucial. Considering the significance of the latent variables, in this study, we evaluated the models with varying number of latent space variables. Additionally, we intended to observe the behavior of the model with respect to the data distribution in the train dataset. The existing studies, with some exceptions, have been conducted on the balanced training dataset of the benchmark data. For a fair comparison, we used a similar configuration of the train and test datasets. However, to evaluate the robustness of the proposed method, we also train it using an unbalanced dataset.

### Effect of latent variables

**Figure 4:**
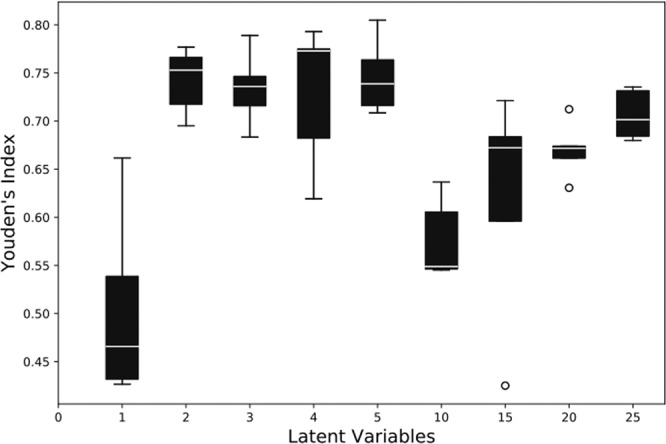
Effect on the Youden’s index values by varying number of variables in the latent space.

**Figure 5:**
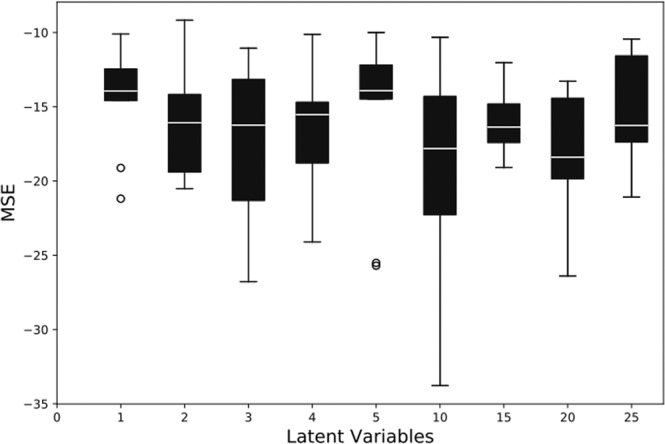
MSE values depicting reconstruction error for various auto-encoder models.

In the first ablation study, we observe the effect of varying the number of variables in the latent space by maintaining a constant balanced data distribution for training. Since the latent space is sparsely populated, it satisfies the limitation on the compression factor. Therefore, we start the evaluation by maintaining the latent space variable of length 25. The latent space variables (LV) are then systematically reduced and evaluated by reducing 5 neurons. Subsequently, after evaluating the performance of the model for LV 5 neurons, the latent space variables were further reduced one by one. For each configuration, 20 simulation runs are performed, and the values of the statistical parameters such as MCC, Youden’s index, balanced accuracy, F1 score, and MSE are observed. The mean values of Youden’s index and the MSE for the reconstruction error have been depicted in Figs. 4 and 5, respectively.

### Effect of data distribution

Another ablation study was performed to observe the sensitivity of the model for training the data distribution. To this end, AFPs and non-AFPs were fused in three distinct subsets having AFP and non-AFP ratios of 1:1, 1:2, and 1:3. Additionally, the effect of the latent space variables on the data distribution was considered; therefore, the training was performed on incremental latent space variables. Yang *et al*. studied the effect of an imbalanced training dataset and it has been reported that their classifier does not comprehend the imbalanced data and classifies most of the samples to the majority class^26^, the results therefore are not appreciable. However, the proposed classifier (AFP-LSE) has the tendency to learn further motif information when the number of training samples is increased. Appreciable values of performance metrics in Table 1, suggests that the performance of the classifier can be improved by utilizing the supplementary information from the negative class. As there is a limitation in the availability of AFP datasets, previous studies have been conducted on a small balanced dataset. Therefore, for a comparison, we report the results of the performance of the classifier trained by using similar configurations.

#### Performance evaluation and comparison with contemporary methods

After an analysis of the results obtained from the ablation study performed to determine the optimal parameters and the size of the latent space, the best model is selected as the classifier for AFP and is named as AFP-LSE. The model is trained with CKSAAP encoded samples with *k* = 8, with the number of latent space variables *LV* = 4 and with 1:1 ratio of training and test datasets. The model is evaluated on an independent test dataset, and its results on the statistical parameters are better than those obtained by the previously reported methods. This study evaluates the performance of the classifier on the parameters reflecting the true efficacy of the classifier by considering the imbalanced condition of the training and testing datasets. Therefore, we emphasize the parameters MCC, balanced accuracy, and Youden’s index due to their insensitivity toward imbalance in classes. The best model showed the MCC value of 0.52, balanced accuracy of more than 90%, and Youden’s index value of 0.81. The performance of AFP-LSE is compared with those of the existing methods as shown in Table 2. Based on the prediction results, AFP-LSE achieved superior performance on all the statistical measures. Particularly, improvements of approximately 2% and 5% in the balanced accuracy and Youden’s index, respectively, were observed when compared with the corresponding values for the best classifier in the literature i.e., CryoProtect^29^. Similarly, the best values of the MCC and F-score were demonstrated by AFP_PSSM^24^, whereas the proposed classifier shows improvements of approximately 52% and 68%, respectively, for the aforementioned parameters.

**Table 2:** Comparison of best performing AFP-LSE model with contemporary approaches on an external validation set containing 181 AFPs and 9193 Non-AFPs and trained with a balanced dataset comprising 300 AFPs and 300 Non-AFPs.

Methods	Classifier	Sensitivity	Specificity	Acc	Youden’s Ind	Bal Acc	MCC	F-Score
iAFP^23^	SVM	13.2%	97.0%	95.3%	0.10	55.1%	0.08	0.10
AFP-Pred^22^	RF	84.6%	82.3%	83.3%	0.63	83.4%	0.23	0.15
AFP_PSSM^24^	SVM	75.8%	93.2%	93.0%	0.69	84.5%	0.34	0.29
AFP-PseAAC^25^	SVM	86.1%	84.7%	84.7%	0.70	85.4%	0.26	0.17
RAFP-Pred^28^	RF	84.0%	91.0%	90.9%	0.75	87.5%	0.33	0.26
CryoProtect^29^	RF	87.2%	88.3%	88.2%	0.76	87.7%	0.30	0.22
AFP-CKSAAP^36^	DNN	94.0%	87.0%	88.0%	0.81	90.5%	0.32	0.22
**Proposed**	AE + DNN	86.7%	93.9%	93.7%	0.81	90.3%	0.52	0.49

#### Prediction of novel AFP candidates

Considering the extreme rarity of AFPs within entire organism proteomes, herein, we perform the screening of novel AFP candidate proteins. An independent dataset containing 10 candidate AFPs was obtained from the INTERPRO^56^ database. The sequences in this independent test dataset were not present in the positive or negative datasets of AFP-LSE. The prediction results of AFP-LSE were compared with those of PSI-BLAST search from UNIPROT^57^ and SWISSPROT^58^ databases on *E* = 0.1. The AFP-LSE predicted 9 proteins as AFPs and only 1 protein is predicted as non-AFP. Interestingly, the same protein is also classified as non-AFP by PSI-BLAST. Compared with AFP-LSE, PSI-BLAST retrieved only 4 out 10 candidate sequences as AFPs as shown in Table 3. The NCBI database annotated 4 out of 10 sequences as hypothetical or unnamed proteins; further three of them were characterized as Type I antifreeze, or AFP-like domain-containing proteins, whereas the annotations of the remaining three are shown in Table 3. The performance of AFP-LSE suggests that it can be effectively utilized for the annotation of hypothetical proteins.

**Table 3:** Prediction results for 10 candidate antifreeze proteins obtained from INTERPRO^56^ database.

GI Number	UniProtKB ACC	AFP-LSE	PSI-BLAST	NCBI Definition
26325086	Q14DU1	Non-AFP	Non-AFP	Kelch-like 11 (Drosophila)
74221639	Q3V0I3	AFP	AFP	Uncharacterized protein
12843602	Q9D7P2	AFP	Non-AFP	Uncharacterized protein
30249105	Q82VH2	AFP	AFP	Type I antifreeze protein
45435722	Q66D88	AFP	Non-AFP	Hypothetical protein
281341260	D2H0G8	AFP	AFP	AFP-like domain-containing protein
2315605	O16596	AFP	Non-AFP	Cell division coordinator CpoB
260817607	C3YJ26	AFP	AFP	AFP-like domain-containing protein
26388908	Q8BMV6	AFP	Non-AFP	RIKEN cDNA E130116L18 gene
26348120	Q8C1R8	AFP	Non-AFP	Uncharacterized protein

## Discussion

**Figure 6:**
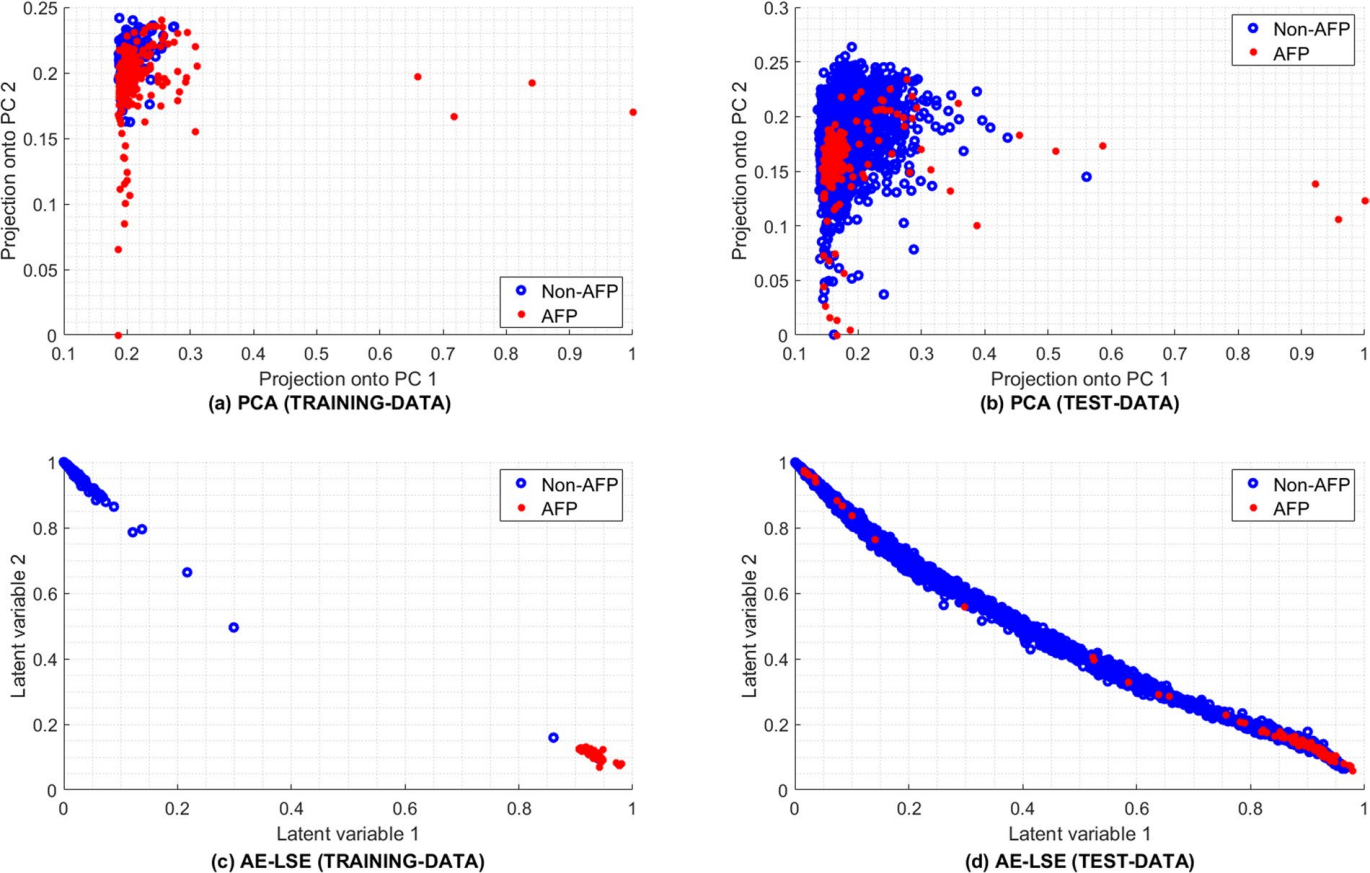
Comparison of proposed auto-encoder-based latent space encoding (AE-LSE) with principal component analysis (PCA) method for 2D projection.

Due to the lack of availability of AFP samples, the nature of the available dataset is skewed, therefore, the classification of AFPs from non-AFPs poses a class imbalance problem which is challenging for machine-learning algorithms^59^. In addition to this class imbalance, there is an issue of rare cases of sub-types in AFP, as in “AFP” class, where only fewer sub-types are in abundance, which leads to intra-class imbalance and introduces outlier artifacts in designing a reliable classifier. In contrast, in typical classification problems e.g., in the case of lysine acetylation sites prediction in proteins, or the identification of protein-protein binding sites, there is an availability of a substantially large number of positive and negative samples in datasets, hence, they do not suffer from the problem of class imbalance or intra-class variation^33,60,61^. Another challenge faced in the classification of AFPs is the variation in the sequences of AFPs, which subsequently produces features with low inter-class and high intra-class variance. These inevitable phenomena are the consequences of the similarity exhibited by AFPs with different protein families from which they are assumed to be evolved^18,19^ and because different AFPs present low sequence similarity among each other. Principal component analysis (PCA) projection of CKSAAP features, which is discussed later in the text, establishes explicit evidence in Fig. 6(a,b), that both AFPs and non-AFPs appear in an overlapping fashion, suggesting that the development of the AFP classifier using linear methods is an arduous task.

For an insightful understanding of CKSAAP representation-based classification of AFPs using the given dataset, we present a comparison of the PCA and AFP-LSE methods. For visual assessments, the data were projected on two dimensions utilizing the top two eigenvectors in the case of PCA and two latent spaces in the case of AFP-LSE. As shown in Fig. 6(c), the proposed non-linear auto-encoder-based latent space encoding (AE-LSE) presents superior learning capabilities and maps the AFPs and non-AFPs in separate regions in contrast to the linear unsupervised sub-space learning method of PCA depicted in Fig. 6(a), which fails to do so, revealing that both classes are inseparable in a linear sense.

The same eigenvectors and the latent space from PCA and AE-LSE respectively, obtained from training are then utilized to project the test data. Differences in the mapping capabilities of AFPs can be observed for both the PCA and AE-LSE methods in Fig. 6(b,d) respectively. It can be observed in the bottom right of the Fig. 6(d) that the AE-LSE method forms clusters of AFP samples. Nevertheless, there is some overlapping of non-AFPs, the overall separability of the data projected through the AE-LSE method is better than that of the data linearly projected by the PCA, indicating that the discovery of unknown groups using PCA is strenuous. This helps in understanding the working principle of the proposed method and the motivation for the development of non-linear auto-encoder-based learning of latent space.

The proposed method can contribute toward the design of a superior mapping function resulting in a reduction of dimensions while retaining the information that separates the AFP from the non-AFP samples. Recently, many researchers have shown interest in auto-encoder-based models^62^. However, to the best of our knowledge, no auto-encoder-based classifier has been proposed for the classification of protein sequences. The proposed model can be used for the prediction of other types of proteins as well, for instance, bioluminance proteins (BLPs)^63^ and extra cellular matrix proteins (ECM)^64^ etc. In particular, it can be utilized for the dimensionality reduction in highly non-linear classification problems where number attributes are higher than the training samples. To avoid overfitting, we used regularization techniques such as dropout and batch-normalization in this study. For future studies we would recommend utilizing transfer learning approach where the AFP-LSE model is first trained with a closely related classification task and later fine-tuned for AFP dataset. However, transfer learning and other training strategies are beyond the scope of this study. The Python implementation of the proposed algorithm has been made public, and interested user can utilize the algorithm for their problem of interest. The algorithm is available at (https://github.com/Shujaat123/AFP-LSE). In the near future, we would like to explore auto-encoder-based classifiers further for other bio-informatics problems.

## Conclusion

The prediction of AFPs due to the unavailability of a substantial dataset and the inherent diversity in the sequence and structures is a challenging classification problem that has been addressed by various researchers. In the proposed prediction method, each protein sequence was encoded using CKSAAP with *k* = 8. The results of our previous study showed that these features can significantly contribute to the classification performance. For classification, we proposed a novel machine-learning-based method for the AFP prediction. The method uses an auto-encoder for feature compression, and these reduced features are used to train the neural-network-based classifier. A comparison of the proposed non-linear mapping method with the linear projection approach of PCA demonstrated superior classification capabilities of the proposed method. A comprehensive ablation study was performed for a better understanding of the effect of latent space variables as well as the impact of training data distribution, and widely used biostatistics nomenclatures were evaluated. The method yields excellent classification results on the benchmark dataset, outperforming the existing methods, particularly yielding an MCC value of 0.52 with a Youden’s index of 0.81.
